# Professional and Volunteer Refugee Aid Workers–Depressive Symptoms and Their Predictors, Experienced Traumatic Events, PTSD, Burdens, Engagement Motivators and Support Needs

**DOI:** 10.3390/ijerph16224542

**Published:** 2019-11-17

**Authors:** Andrea Borho, Ekaterini Georgiadou, Theresa Grimm, Eva Morawa, Andrea Silbermann, Winfried Nißlbeck, Yesim Erim

**Affiliations:** 1 Department of Psychosomatic Medicine and Psychotherapy, University Hospital of Erlangen, Friedrich-Alexander University Erlangen-Nürnberg (FAU), Schwabachanlage 6, 91054 Erlangen, Germany; andrea.borho@uk-erlangen.de (A.B.); ekaterini.georgiadou@klinikum-nuernberg.de (E.G.); grimm_theresa@web.de (T.G.); eva.morawa@uk-erlangen.de (E.M.); andrea.silbermann@uk-erlangen.de (A.S.); winfried.nisslbeck@uk-erlangen.de (W.N.); 2 Department of Psychiatry and Psychotherapy, Paracelsus Medical University Nürnberg, Prof.-Ernst-Nathan-Str. 1, 90419 Nürnberg, Germany

**Keywords:** Germany, refugee aid workers, depression, traumatic experiences, PTSD, burdens, engagement motivators, support needs, trainings

## Abstract

In 2016, the Department of Psychosomatic Medicine and Psychotherapy of the University Hospital of Erlangen started conducting training for professional and voluntary aid workers. In total, 149 aid workers took part in the training courses, of which 135 completed the corresponding questionnaires. Engagement motivators, perceived distress in refugee work and training needs were examined. Moreover, depressive symptoms, the prevalence of traumatic experiences and symptoms of posttraumatic stress disorder were explored. Participants named helping others as the highest motivating factor for their work with refugees and communication problems as the main burden. Thirteen aid workers (10.1%) showed clinically relevant depressive symptoms. In total, 91.4% of refugee aid workers had experienced at least one traumatic event personally or as a witness but only three (3.6%) fulfilled the psychometric requirements of a PTSD diagnosis. These three participants all belonged to the professional aid workers (6.3%). More severe symptoms of depression were significantly associated with female gender (β = 0.315, *p* = 0.001), higher perceived burdens of refugee work (β = 0.294, *p* = 0.002), and a larger number of experienced traumatic events (β = 0.357, *p* < 0.001). According to our results, we recommend psychological trainings and regular screenings for psychological stress in order to counteract possible mental illnesses.

## 1. Introduction

Today, massive displacement and refugee movements are increasing social problems [1]. Especially the integration and inclusion of asylum seekers is a permanent challenge for the European societies [2].

At the peak of the European refugee crisis in 2015 and 2016, more than 2.6 million asylum seekers were registered in the European Union, with 1.2 million registrations in Germany alone, more than ever before [3]. With an asylum application number of 476,649 in Germany in 2015, this corresponds to an increase of 135% compared to the previous year and in 2016 this number rose again by 63.5% compared to 2015 [4,5].

That sharp rise in the number of refugees in Germany and Europe in the summer of 2015 temporarily overwhelmed public administration for the registration, reception, accommodation, and care of refugees [6]. The resulting increased need for professional and volunteer refugee helpers could fortunately be covered by a huge number of citizens who started engaging in refugee aid work [7]. In 2017, a representative population survey with 1.387 participants was commissioned by the German Federal Ministry of Family Affairs using face-to-face interviews. According to their results, around 14% of the German citizens aged 16 and older have provided at least temporarily active help for refugees since their increased arrivals in 2015. Out of these, 36% volunteered for the first time due to their commitment to refugees. Compared to the total population, volunteers in refugee aid tend to be better educated. In addition, they are more likely to have a migration background than in other areas of social work (25% vs. 15%) [2,6].

But what drives so many people to get engaged in refugee work? Both altruistic as well as self-serving motives seem to play a role. In a Germany-wide online study with 2291 voluntary refugee helpers, altruistic motivation was identified as the main reason for their engagement. For 85.5% of the refugee helpers it was a great motivation to help the weaker due to their commitment [7]. In the study by the German Federal Ministry of Family Affairs, all participants who were active in refugee aid at the time (11% of the total sample) were interviewed again in more detail. It turned out that one third (34.6%) of volunteer refugee aid workers had a rather “self-serving” motive for their engagement (e.g., recognition for their work, useful for career). Nevertheless, it should be noted that two thirds focused on altruistic motives [2]. The desire to help others plays a particular role slightly more frequently for refugee aid workers than for volunteers in other areas [6,7]. Refugee aid workers assume that their commitment will make a difference and that their contribution will be needed [6]. However, it should be noted that all these mentioned studies have used self-assessment questionnaires which may bias the results by social desirability [8].

Even though refugee aid workers seem to find an important meaning in their work, they often struggle with the conditions of their work [9]. They are complaining about high job demands, high workload, communication problems, permanent changes in social policies and legislation, limited financial resources, major role conflicts, limited control, and low decision latitude [7,10,11,12]. In a study with German volunteer refugee aid workers, 9% found themselves burdened by a lack of recognition and appreciation in their voluntary work [2]. Some qualitative studies also found that staff perceived unrealistic demands from clients and encountered violent and aggressive behavior by clients as distress factors [13,14].

Another major challenge that is special for refugee aid workers is to deal with the suffering of refugees and to regularly hear about their serious traumatic histories [12]. It is already well documented that professionals working with traumatized clients are susceptible to secondary traumatic stress, also described as vicarious traumatization or compassion fatigue [15,16,17]. Refugees represent a highly vulnerable client group, often suffering from traumatic experiences, posttraumatic stress disorder, and depression [18,19,20,21,22,23]. Taking a closer look at the mental health of refugee aid workers themselves, a German national cross-sectional online study from 2018 revealed that 17.3% of the 1712 participating refugee aid workers were positively screened for depression, 57.4% had experienced at least one traumatic event, and comparable to the general German population 2.8% had a positive PTSD screening [9]. In a National Epidemiologic Survey of Goldstein et al. (2016) [24] in the United States, 68.6% of the 36.309 respondents reported having experienced at least one traumatic event during their lifetime and 6.1% were positively screened for PTSD. Even if these figures are higher than in the German study, this suggests that only a small percentage of individuals develop PTSD after experiencing traumatic events.

Considering the factors which are related to psychological stress of aid workers, a study with humanitarian workers in South Sudan estimated that chronic stress at work was positively associated with depression [25]. Different studies found that the length of experience in refugee work was a protective factor among professional refugee service providers being associated with fewer mental health problems such as emotional exhaustion [16]. Besides that, more hours worked per week were associated with higher compassion satisfaction in refugee caregivers [26]. Pell (2013) reported that the strongest effect on mental symptoms among refugee helpers was caused by their own traumatic history [19]. Nevertheless, to date little is known about the specific association between job demands and mental health problems, such as depressive symptoms, among refugee aid workers [12].

Variations in the level of psychological distress between psychologically trained aid workers and untrained aid workers have been found [13,27,28]. A study with 115 refugee aid workers indicated that psychologically trained helpers had significantly lower burnout values and somatic symptoms as the compared untrained aid workers [19]. That result underlines the importance of psychological trainings for refugee helpers. On the one hand, these trainings should convey knowledge about how to deal with traumatized refugees and, on the other hand, provide strategies for maintaining one’s own mental health [9,13,29].

The Department of Psychosomatic Medicine and Psychotherapy at the University Hospital of Erlangen recognized these needs of refugee aid workers and started to get in contact with refugee helpers and aid organizations in 2015. Within these relations, the researchers developed a psychological training on the mentioned topics. As part of these training sessions, they designed a naturalistic study in the form of a questionnaire survey. It was carried out to gather refugee aid worker’s sociodemographics, engagement motivators, specific burdens in refugee work and needs. Additionally, they were screened for experienced traumatic events, possible PTSD symptoms and severity of depressive symptoms as well as examined for their related predictors. In order to identify eventual differences between professional aid workers (PAD) and voluntary aid workers (VAD), those factors were analyzed separately for these two groups. This study is characterized by the combination of the assessment of both motivational factors and psychological stress. In contrast to previous online studies on this topic, this extensive and naturalistic study design can create a much more complete picture of these people.

## 2. Materials and Methods

In 2017, Grimm et al. already published preliminary information about the engagement motivation, perceived distress at refugee work, and needs for support of 45 participants of the here presented study sample [30]. In addition, depressive symptoms as well as the prevalence of traumatic experiences were assessed. Among these refugee workers, only two (4.4%) showed an elevated score for depressive symptoms. However, 93.3% of participants had already experienced at least one traumatic event personally or as a witness.

Due to the small sample size and to verify the detected results, the researchers decided to extend the study sample, as they continued to deliver further training sessions on demand of communities and aid workers. Furthermore, additional information on PTSD symptoms and predictors of depressive symptoms were analyzed to provide a more detailed picture of this sample.

### 2.1. Participants

A total of 149 aid workers took part in the training courses, of these 135 completed the questionnaires. This corresponds to a response rate of 90.6%. The 90 PAD and 45 VAD were recruited in Bavaria in collaboration with the Erlangen city council, the district administration of Ansbach, the German Samaritan Workers Union (ASB, German charitable aid agency), and several refugee helper networks. The training as well as several items of the questionnaire were developed for adults working with refugees in asylum seekers’ shelters or in other contexts, for example as their teacher, interpreter or social education workers. As depicted in the methods section, validated and well established psychometric questionnaires were employed to measure depression and posttraumatic symptoms.

### 2.2. Procedure and Setting

From January 2016 to January 2019 eight in-person trainings on migration and its effects on mental health were organized by the Department of Psychosomatic Medicine and Psychotherapy of the University Hospital of Erlangen. Main contents of this training were: migration and trauma-related mental disorders, symptoms of posttraumatic stress disorder, practical help in difficult situations, and self-help strategies for aid workers.

In addition to convey this content, participants were asked to fill in the provided questionnaires. In total, the training lasted about four to six hours.

This study was conducted in accordance with the Declaration of Helsinki, and the protocol was approved by the Ethics Committee of the Medical Faculty of the University of Erlangen-Nürnberg (Project identification code: 72_17 B). All subjects gave their informed consent for inclusion before they participated in the study.

### 2.3. Measures

#### 2.3.1. Sociodemographic, Migration-Specific and Aid Work related Variables

In addition to sociodemographic data, participants with migration background were asked to answer several further questions, e.g., about their length of residence in Germany or their German language skills. To identify persons with migration background, the current definition was used: “A person has a migration background if s/he or at least one of his/her parents did not acquire the German citizenship at birth” [31].

With regard to the aid work related variables, a self-developed questionnaire was used to assess the fields of activity and burdens in refugee work, needs of the aid workers, but also motivational factors for their work.

To answer the question about their burdens at work, the participants were given seven answer alternatives to choose from (e.g., communication problems, lack of financial resources, aggressive behavior of refugees). Another item (“What is your motivation for working with refugees”) should record the motivational factors on the basis of three possible answers (helping others, religious values, financial reasons). For both items, multiple answers could be chosen. It was also possible to specify one’s answer in plain text. On the one hand, the item “How burdened do you feel in working with refugees?” assessed the overall burden of working with refugees on a 10-point Likert scale from 1 = not at all to 10 = totally and on the other hand, using the same 10-point Likert scale, participants were asked to what degree they experienced working with refugees as personal enrichment.

The participants needs for support were determined by eight questions (e.g., “I want to learn how to take good care of myself in view of the stress in working with refugees”, “I need information about possible trauma-related mental problems after traumatic experiences”) on a 5-point Likert scale (−2 = not at all, +2 = yes, exactly). Again, multiple answers as well as the possibility to add their own content in plain text were given.

#### 2.3.2. Traumatic Events and PTSD

The occurrence of traumatic events and symptoms of posttraumatic stress disorder were assessed by the self-rating questionnaire Essen Trauma-Inventory [32]. This screening instrument assesses potentially traumatizing events and questions concerning objective and subjective threat of life (criterion A1 and A2). In addition, it covers key characteristics of PTSD—intrusion, avoidance and hyperarousal as well as dissociation in corresponding subscales. With beginning of the first training session, all participants marked “yes” or “no” in the trauma list of the questionnaire if they had personally experienced and/or witnessed one or more of the listed traumatic events. After a preliminary analysis of the first three training sessions, a very high number of traumatic experiences among the aid workers was observed. In order to detect possible hints for PTSD, from the fourth training session onwards, the participants were additionally asked to answer the questions about the A1- and A2 criterion. They also rated the 23 items on the PTSD symptom list on a 4-point Likert scale that ranged from “never” (0 points) to “very often” (3 points). Clinically apparent PTSD is indicated if a participant has experienced at least one traumatic event, if s/he meets the A1 and A2 criteria, and if the total score for the PTSD symptom list (intrusion, avoidance and hyperarousal) reaches a cut-off value of 27. Cronbach’s alpha in the present study sample was 0.91.

#### 2.3.3. Depressive Symptoms

For the assessment of depressive symptoms, the German version of the depression module of the Patient Health Questionnaire (PHQ-9) [33,34] was used. This self-report instrument includes items based on DSM-IV criteria of major depressive disorder [35] describing the somatic and cognitive-emotional symptoms of depression. These items refer to problems within the previous two weeks. With the number of nine questions, this instrument is comparatively short, but has high sensitivity (80%) and specificity values (92%) [36]. Each item is rated on a four-point Likert-scale ranging from 0 (not present at all) to 3 (present nearly every day). The total score ranges from 0 to 27 points and from a total score of 10 points, moderate levels of depressive symptoms can be assumed [34]. For the current study, Cronbach’s alpha was 0.85.

#### 2.3.4. Attitude Structure of Volunteers

The German Scales of the Attitude Structure of Volunteers (SEEH) capture eight motivational aspects for voluntary work that can be assigned to the two dimensions “self-serving orientation” and “altruistic orientation” [37]. The self-serving orientation includes the following scales: social commitment, self-experience, self-esteem, social influence, job balance and career. The altruistic orientation includes the scales of social responsibility and political responsibility. Answers are scored on a nine-point scale, with higher scores corresponding to greater importance of the query aspect as a source of voluntary engagement. This questionnaire is designed especially for volunteers. Therefore, only the VAD of the presented sample were asked to complete it. In this study, Cronbach’s alpha for the dimension “self-serving orientation” was 0.84 and for “altruistic orientation” 0.96.

### 2.4. Statistical Analysis

Data analyses were conducted using SPSS Version 21 (IBM Corporation, Armonk, NY, USA). We used multiple imputation via fully conditional specification to address missing values in our dataset. Predictive mean matching was used for all imputation models to ensure plausible imputations. In the PHQ–questionnaire, four participants had one missing item (3.0%), in the ETI–questionnaire five participants had one missing item, one participant had two and one had three missing items (5.2%) and in the SEEH–questionnaire one participant had one missing value, two participants had two and two participants had four missing values (11.1%).

For descriptive purposes, mean values, standard deviations, ranges, and frequencies were calculated. Differences between volunteer aid workers and professional aid workers were evaluated with two-sampled t-tests and χ²-tests. In case of non-normal distributions, Mann-Whitney-U-tests were calculated instead. Fisher’s exact test was used if the expected count in more than 20% of cells was less than five. To analyze which sociodemographic and work-related variables predict the severity of depressive symptoms, a multiple linear regression analysis with enter method was conducted. Prior to this analysis, to avoid multicollinearity, bivariate correlations were computed among the included variables. There were no correlation coefficients higher than 0.70 so that none of the tested variables had to be excluded from the multiple regression model. For all analyses, the level of significance was predetermined at *p* ≤ 0.05.

## 3. Results

### 3.1. Sociodemographics

The sociodemographic characteristics of the participants are presented in Table 1. We revealed group differences in sociodemographic parameters for education (χ^2^ = 17.294, *p* = 0.004), employment status (χ^2^ = 71.242, *p* < 0.001), and migration background (χ^2^ = 7.951, *p* = 0.005). PAD were higher educated, more often full-time employed, less likely to be unemployed, and had more often a migration background than VAD.

From the total sample, 39 participants (28.9%) had a migration background. Out of these persons, 22 are German native speakers born in Germany who were classified in that group because of their parents’ immigration history (see official definition cited above). At the time of data collection, the average length of residence in Germany for the other 17 participants with a migration background was 18.7 years (SD = 12.3 range: 4–45 years). Of these persons, 11 (64.7%) rated their German language skills as very good and six (35.3%) as good. The countries of origin were very diverse. The most frequently mentioned countries of origin were Turkey, Egypt, and Ukraine with two persons each.

### 3.2. Information on Refugee Work of the Study Sample

While, most PAD worked with refugees in schools or counseling centers (48.9%), the majority of VAD were active in the field of everyday support and daily care (44.4%). Detailed information about the work activities of refugee aid workers is given in Table 2. A significant difference between the two groups was found in the weekly hours spent on refugee work (t = −6.291, *p* < 0.001).

### 3.3. Motivation

**Figure 1:**
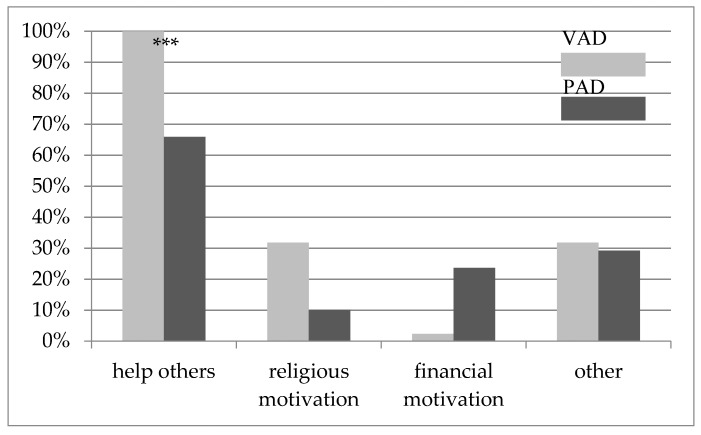
Engagement motivators for refugee work for voluntary aid workers (VAD) and professional aid workers (PAD). Multiple answers possible; others = e.g., this is my job; intercultural interest; *** *p* < 0.001.

As pictured in Figure 1, helping others proved to be the highest motivator for working with refugees. However, VAD reported this reason significantly more often than PAD (χ^2^ = 19.01, *p* < 0.001). Other reasons mentioned by participants were e.g., social responsibility or cultural interest.

As personal enrichment through the work, the total study sample reported on a scale from 1 to 10 an average value of 7.54 (SD = 2.07), VAD had an average value of 8.16 (SD = 1.49), and PAD an average value of M = 7.23 (SD = 2.25). The perceived enrichment was significantly higher for VAD than for PAD (t = 2.48, *p* = 0.006).

### 3.4. Specific Engagement Motivators of VAD

**Figure 2:**
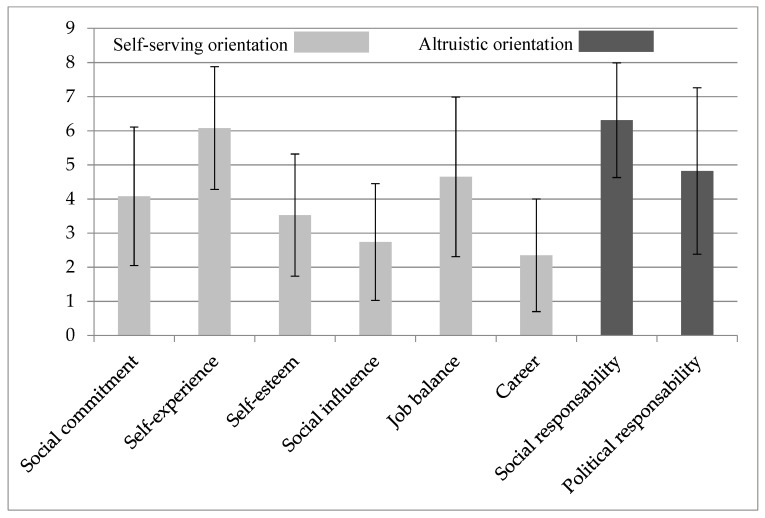
Special engagement motivators for refugee work of VAD. Scale from 1 = absolutely irrelevant to 9 = absolutely relevant.

The engagement motivators measured with SEEH are shown in Figure 2. For VAD the most significant engagement motivators for refugee aid were self-experience (M = 6.06, SD = 1.80) and social responsibility (M = 6.31, SD = 1.68). The average altruistic orientation of the VAD was 5.63 (SD = 1.79) and the average self-serving orientation was 4.05 (SD = 1.22).

### 3.5. Burdens

The average perceived burden of working with refugees was 4.56 (SD = 2.28) for the total sample. PAD (M = 5.00, SD = 2.31) experienced this kind of work as significantly more stressful than VAD (M = 3.67, SD = 1.97; t = −3.23, *p* = 0.002).

**Figure 3:**
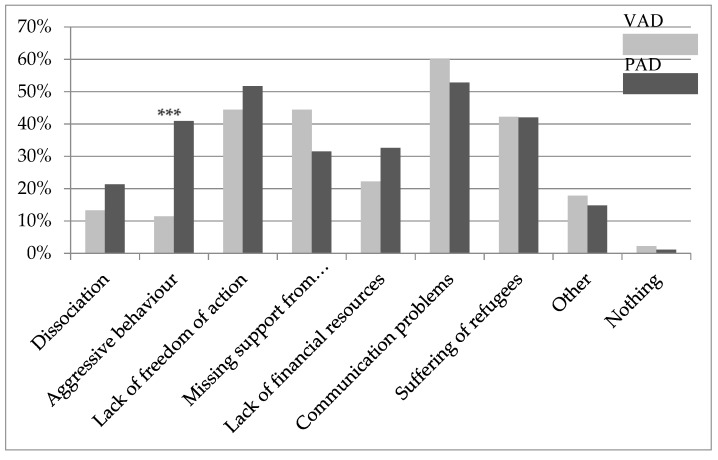
Burdens in refugee work for VAD and PAD. Multiple answers possible; other = e.g., this is my job; intercultural interest; *** *p* < 0.001.

Looking at the individual stress factors in refugee work, there was only one significant difference between PAD and VAD in the area of dealing with aggressive behavior of refugees (χ^2^ = 11.96, *p* = 0.001). With respect to the other factors shown in Figure 3, the two groups did not differ significantly.

### 3.6. Needs

The participants showed the greatest need in recognizing mental problems of the refugees and being able to help refugees if they are mentally distressed. Detailed information about the needs of VAD and PAD are provided in Table 3.

### 3.7. Depressive Symptoms

With a total PHQ-9 score of at least 10 points, 13 participants (10.1%) were screened as positive for at least moderate depressive symptoms. The total score for depressive symptoms in this sample was 4.16 (SD = 3.89). Looking at the two groups individually, four participants (8.9%) of the VAD and nine participants (10.0%) of the PAD showed elevated depressive symptom manifestations. In terms of their mean values, there was no significant difference between the groups (VAD: M = 4.28, SD = 4.11; PAD: M = 4.10, SD = 3.80; t = 2.23, *p* = 0.82).

### 3.8. Traumatic Events and PTSD

A total of 117 participants (91.4%) had personally experienced and/or witnessed traumatic events (40 of the VAD (90.9%) and 77 of the PAD (91.7%)). 81 persons (60%) experienced at least one trauma personally, 98 (72.6%) as a witness, and 10 (7.4%) personally and as a witness. Considering only the VAD, the distribution turned as followed: 31 persons (68.9%) personally, 29 (64.4%) as witness, and five (11.1%) personally and as witness. For the PAD the results were: 50 persons (55.6%) personally, 69 (76.7%) as witness, and five (5.6%) personally and as witness. In this respect, there was no significant difference between VAD and PAD. The lifetime prevalence of specific traumatic events is presented in Table 4.

On average, the participants reported having experienced and/or witnessed M = 2.56 traumatic events (SD = 1.88, range: 0–8). For VAD the mean value was M = 2.19 (SD = 1.65, range: 0–8) and for PAD it was M = 2.76 (SD = 1.98). Regarding the traumatic events, there were no significant differences between the two groups. Three (3.6%) of the 84 participants (training session four to eight8) who completed the entire ETI questionnaire fulfilled the psychometric requirements of a PTSD diagnosis. These three participants all belonged to the 48 PAD (6.3%).

### 3.9. Predictors of Severity of Depressive Symptoms

A multiple linear regression was calculated to examine the influence of several sociodemographic variables, refugee work related variables, and the amount of experienced traumatic events on the severity of depressive symptoms (PHQ total score) of the total sample. For depression symptoms a significant regression equation was found (F(6,104) = 5.042, *p* < 0.001), and the explanation of variance was 22.5%. As can be seen in Table 5, more severe symptoms of depression were significantly associated with female gender, higher perceived burdens of refugee work, and a larger number of experienced traumatic events.

## 4. Discussion

Using a sample of 135 refugee aid workers, the main goal of the present study was to investigate their engagement motivators, burdens in refugee work, specific needs, and to screen for their experienced traumatic events, resulting PTSD symptoms, and depressive symptoms. The striking findings of our study were the exceptionally high degree of trauma exposure and the low levels of psychopathology among our sample. In total, 91.4% of the participating refugee aid workers had already experienced at least one traumatic event but only three (3.6%) fulfilled the psychometric requirements of a PTSD diagnosis. These three participants all belonged to the professional aid workers (6.3%). Despite high reported load factors, such as communication problems with refugees, only 10.1% showed elevated depressive symptoms.

Regarding the sociodemographic characteristics of the total sample most of the aid workers were female (65.4%) and middle-aged (M = 44.1), which is in line with other surveys according to which women are much more likely to become active in voluntary work [7,9]. It was remarkable that 73.3% of the participants had a university degree, considerably more than in the general population in Germany (17.6%) [38]. However, our result is comparable to the study commissioned by the German Federal Ministry of Family Affairs, according to which refugee aid workers have a higher level of education than the overall population [2,6]. Especially among the PAD, this percentage was very high (82.2%). This can be explained by the fact that most of the mentioned professions (e.g., teacher, social educator) require a university degree. Migration background was found in 28.9% which is slightly higher than in the German general population (23.6%) [39]. That percentage is still in line with previously found study results [2,6]. In the present study the proportion of participants with migrant background was significantly higher among the PAD (36.7%) than among the VAD (13.3%). Earlier surveys have already shown comparable results for volunteers [2,9]. The high proportion of immigrants among the PAD may be linked on one side to the high level of intercultural competences they have due to their own migration background. On the other side, migrants may have experienced integration impediments themselves and may choose aid activities by phenomenon of identification or empathy. Thus, they were in great demand on the labor market at the beginning of the refugee crisis.

Returning to one of our initial questions on the motivational factors of refugee work, we revealed that every VAD and 66% of the PAD wanted to “help others”. Although the two groups differed significantly on this point, it was still the most frequently named motivational factor of both groups. Financial motives were mentioned by 23% of the PAD and only 2% of the VAD. These results suggest a rather altruistic motivation for refugee work and confirm earlier studies with similar results [2,6,7]. Overall, both groups felt greatly enriched by working with refugees.

We took a closer look at the motivational factors of those participants who engaged in aid work voluntarily. The evaluation about the attitude structure of volunteers (SEEH-questionnaire) confirmed the assumption of a more altruistic orientation of the volunteers, with self-serving factors such as “self-experience” also having a not negligible impact. These results confirm previous findings according to which the motives of VAD cannot be clearly assigned only to altruistic or to self-serving orientation [2,40].

As shown in earlier surveys, the present study confirms that refugee aid workers are exposed to numerous stressors such as communication problems (due to language difficulties) or lacking freedom of action (e.g., due to laws or organizational restrictions) [7,12,41]. In contrast to VAD, PAD suffer significantly more often from aggressive behavior by refugees. This may be related to the fact that PAD are often in the role of decision-makers with a certain power over the refugees (e.g., as teachers, security personal), whereas volunteers generally offer support to refugees at the same hierarchical level. Overall, PAD felt more affected by working with refugees than VAD did.

In our sample, we detected the greatest need for support in “identifying psychological problems of refugees” and in “being able to help them if they are mentally distressed”. Especially VAD were interested in learning helpful strategies for their daily work. For PAD it was a bigger need to receive psychosocial support for themselves than for VAD. This again shows that the voluntary nature of working with refugees seems to have an influence on mental well-being. These results underline the postulated demand to impart knowledge about the following topics to the aid workers: How to deal appropriately with traumatized and vulnerable people, how to raise awareness of potential threats to one’s own mental health and which strategies are useful for its preservation [13,27].

Despite the large load factors, we found positive depression screening in only 10.1% of all aid workers. That value is comparable to that of the German general population (8.1–10.1%) [42,43] and is much lower than in other surveys about refugee aid workers (e.g., 17.3% [9] or 19.5% [44]). Our sample consisted of many teachers, social pedagogues, or employees of counselling centers. In these professions, basic psychological knowledge and a better understanding of mental disorders can be assumed. For this reason, it can be expected that persons in these groups are able to prevent depressive symptoms to a certain extend. Besides, a major group of our sample started to work with the refugees only a few months before study conduction. This may explain why hints of burnout and exhaustion may not be overdetermined.

As significant predictors of the severity of depressive symptoms, we identified female gender, higher perceived burdens of refugee work, and a larger number of experienced traumatic events. Compared to previous findings, there is consistent evidence from epidemiological and clinical studies that female gender is associated with increased odds of experiencing depression [45]. High perceived burdens of work can cause chronic stress, which in turn may lead to depression and other mental illnesses [46]. Regarding traumatic events, a study from 2016 about migrant populations revealed that having experienced traumatic events in the past increases the likelihood to common mental disorders such as depressive symptoms [47].

Regarding the experienced traumatic events, 91.4% of the participants experienced at least one traumatic event personally and/or as a witness in the past or during their work in refugee aid. Compared to the German general population, in which the lifetime prevalence for traumatic experiences is about 24%, this is an extremely surprising result [48]. Our percentage is even very high in comparison to the study by Jobst et al. (2018) in which 57.4% of the participating German refugee aid workers have already experienced a traumatic event [9]. One possible explanation would be that the aid workers make traumatic experiences while working with refugees (e.g., securities in accommodation centers). In the PAD group it may be partly explained by the slightly above-average number of people with immigration history in our study sample who are known to have experienced traumatic events more often than people without migration background [49]. Tagay et al. reported that 53.3% of Turkish-speaking immigrants had experienced at least one traumatic event in their lifetimes, either personally or as a witness [50]. In addition, it is conceivable that people who had already personally experienced traumatization may be more willing to get involved in refugee work and help other traumatized people. Based on their own traumatic experiences, these aid workers can empathetically identify with the refugees. This empathic identification again demonstrates the increased risk for secondary traumatization.

However, looking at the participants’ data on PTSD symptoms, it turned out that although over 90% of the participants reported at least one traumatic experience, only 3.6% also met the criteria of a PTSD diagnosis. Compared to the German population with 2.3%, this figure is only slightly higher [51]. However, compared to the results of the National Epidemiologic Study by Goldstein et al. (2016) [24] with 6.1% in the United States, the figure found in this study is considerably lower. After a more detailed analysis, it became clear that all persons with PTSD were PAD. On one hand, this supports the conclusion already shown that PAD, who work with refugees as part of their job, need psychosocial support. On the other hand, the fact that no one among the VAD had a PTSD diagnosis provides evidence of a higher resilience of these individuals.

### Strengths, Limitations and Implications for Policy and Practice

A major strength of this study is the inclusion of both professional and volunteer refugee aid workers and their comparison. In addition to existing results, our study provides a more nuanced picture of the current situation of refugee aid workers in Germany as we included not only scales on motivation and orientation of aid workers but also on depression and posttraumatic stress disorder. There are also some limitations to our study. Due to the small sample size, we cannot guarantee for representativeness for the entire community of PAD and VAD. As our study is based on self-report assessments, the presented results could be biased by social desirability. In addition, self-rating scales do not fulfill the golden standard of structured interviews. In this respect, it is quite possible that not all psychiatric comorbidities were detected. In literature, we find remarks on drug abuse as self-healing experiments of the concerned people [52,53]. Therefore, future studies should screen for a broader spectrum of mental problems and ideally also for more protective and risk factors. Furthermore, particular attention should be paid to traumatic experiences and PTSD. As the aid worker showed little clinical impairment, sources of their resilience should be investigated. A major group of aid workers in our study recently switched to refugee aid from other fields of social work. Follow-up studies should shed light on the career experiences and development of mental health. In addition, comparative studies with other groups of professionals should examine whether refugee aid workers are exposed to heavier stress than others.

As desired by those affected, based on our results several implications can be derived at a political and organizational level. Despite the good mental health of aid workers this study has established, training should be offered regularly, as aid workers consider information to be an important resource. In a next step, pre-post tests should be conducted to investigate the effect of such trainings on the psychological well-being of refugee aid workers.

Finally, to avoid one of the most frequently mentioned problems, the communication problems, a pool of interpreters should be available.

## 5. Conclusions

Even though the number of refugees has slightly declined in the last years, large numbers of people will be evicted and will move to European countries due to worldwide climatic changes and numerous political crises [54]. With the current study we presented that working with refugees can be a great enrichment but at the same time a huge challenge for aid workers. We demonstrated that refugee aid workers are exposed to numerous stressors. Supervisions and trainings have to be provided to maintain the high motivation of the employed and voluntary refugee aid workers and to support them regarding the specific stress factors of refugee work. Despite the high prevalence of traumatic events experienced in the past, there was an average occurrence of depression among our participants and only PAD showed an above-average frequency of PTSD symptoms. Longitudinal studies on the subject of mental health and it’s protective and risk factors are necessary to investigate this phenomenon. According to the presented results, aid workers are exposed to heavy stress, which pose a risk for mental illness and secondary traumatization. For this reason, we recommend psychological trainings and regular screenings for psychological stress in order to be able to counteract possible mental illnesses at an early stage. 

## Figures and Tables

**Table 1:** Sociodemographic characteristics of the study sample.

Sociodemographic Variables		Total (*N* = 135)	VAD (*n* = 45)	PAD (*n* = 90)	*p*-Values
Age	mean (SD) range	44.1 (14.5) 19–80	48.2 (19.0) 19–80	42.2 (11.3) 23–71	*p* = 0.060 ^a^
		***N* (%)**	***n* (%)**	***n* (%)**	
Gender	Male Female	47 (34.6) 88 (65.4)	14 (30.2) 31 (69.8)	33 (36.7) 57 (63.3)	*p* = 0.523 ^b^
Marital status	Single Married Divorced In a relationship	32 (24.1) 56 (42.1) 19 (14.3) 26 (19.5)	10 (22.7) 20 (45.5) 6 (13.6) 8 (18.2)	22 (24.7) 36 (40.4) 13 (14.6) 18 (20.2)	*p* = 0.941 ^b^
Education	Middle school or less High-school diploma University degree	16 (11.9) 20 (14.8) 99 (73.3)	8 (17.8) 12 (26.7) 25 (55.6)	8 (8.9) 8 (8.9) 74 (82.2)	*p* = 0.004 ^b^
Employment status	Full-time Part-time Unemployed (housewife, pensioner) School/University Studies	70 (51.9) 33 (24.4) 19 (14.1) 13 (9.6)	5 (11.1) 11 (24.4) 18 (40.1) 11 (24.4)	65 (72.2) 22 (24.4) 1 (1.1) 2 (2.2)	*p* < 0.001 ^b^
Religion	Roman-Catholic Protestant Muslim Other None	37 (27.6) 53 (39.6) 7 (5.2) 8 (6.0) 29 (21.6)	12 (27.3) 22 (48.9) 0 (0.0) 4 (9.1) 6 (13.6)	25 (27.8) 31 (34.4) 7 (7.8) 4 (4.4) 23 (25.6)	*p* = 0.083 ^c^
Migration background	Yes No	39 (28.9) 96 (71.1)	6 (13.3) 39 (86.7)	33 (36.7) 57 (63.3)	*p* = 0.005 ^b^

^a^ Mann-Whitney-U test used because of non-normal distribution, ^b^ χ^2^-test, ^c^ Fisher’s exact test.

**Table 2:** Information on refugee work of the study sample.

Refugee Work		Total (*N* = 135)	VAD (*n* = 45)	PAD (*n* = 90)	*p*-Values
		*N* (%)	*n* (%)	*n* (%)	
Field of activity in refugee work	School ^a^ Daily support/care ^b^ Counseling ^c^ Interpreter Accomodation ^d^ Job centre/City council Medical supplies Therapeutic support ^e^ No data	21 (15.6) 23 (17.0) 31 (23.0) 9 (6.7) 13 (9.6) 10 (7.4) 4 (3.0) 4 (3.0) 20 (14.8)	1 (2.2) 20 (44.4) 7 (15.6) 3 (6.7) 2 (4.4) 0 (0) 2 (4.4) 1 (2.2) 9 (20.0)	20 (22.2) 3 (3.3) 24 (26.7) 6 (6.7) 11 (12.2) 10 (11.2) 2 (2.2) 3 (3.3) 11 (12.2)	*p* = 0.131
Weekly working hours ^f^	mean (SD) range	19.4 (17.1) 0–60	6.6 (9.7) 1–40	26.9 (16.0) 0–60	*p* < 0.001
Duration of refugee work ^g^	mean (SD) range	45.9 (82.8) 0.5–360	32.7 (54.7) 1–216	54.8 (96.9) 0.5–360	*p* = 0.208

^a^ teacher, social educator, school psychologist; ^b^ family care, organizing leisure activities, accompaniment (at authorities); ^c^ legal advice, asylum social counseling; ^d^ organization, security in accommodation centers; ^e^ music therapy, psychological support; ^f^ refugee work; ^g^ in months.

**Table 3:** Information on support needs of the total study sample, VAD and PAD.

Support Needs	Total (*N* = 135)	VAD (*n* = 45)	PAD (*n* = 90)	*p*-Values ^a^
	M (SD)	M (SD)	M (SD)	
I want to learn how to take good care of myself in view of the stress in working with refugees.	2.12 (1.07)	2.16 (1.11)	2.09 (1.07)	*p* = 0.787
I would like to be able to help the refugees if they are obviously mentally distressed.	1.48 (0.75)	1.25 (0.44)	1.60 (0.85)	*p* = 0.016
I would like to recognize if a refugee has mental problems.	1.30 (0.62)	1.16 (0.53)	1.38 (0.65)	*p* = 0.015
I need information about possible trauma-related mental problems after traumatic experiences.	1.60 (0.89)	1.55 (0.86)	1.62 (1.67)	*p* = 0.551
I need information on dealing with possible complaints of trauma.	1.63 (0.85)	1.55 (0.82)	1.67 (2.14)	*p* = 0.827
I would like to learn more about the cultural background of refugees.	2.00 (1.03)	1.73 (0.82)	2.14 (1.11)	*p* = 0.042
I need psychosocial support because of the burdens of work with refugees.	3.31 (1.23)	3.63 (1.14)	3.16 (1.25)	*p* = 0.037
I need information about asylum law	2.98 (1.27)	3.12 (1.22)	2.91 (1.30)	*p* = 0.371

Scale from 1 = yes, exactly to 5 = not at all; ^a^ Mann-Whitney-U-Test because of non-normal distribution.

**Table 4:** Lifetime prevalence of traumatic events of the study sample.

Personally and/or as Witness Experienced Traumatic Events	Total (*N* = 135)	VAD (*n* = 45)	PAD (*n* = 90)
	*N* (%)	*n* (%)	*n* (%)
War effort/Military conflict	10 (7.4)	2 (4.4)	8 (9.0)
Prisoner/hostage	7 (5.2)	2 (4.4)	5 (5.6)
Torture	2 (1.5)	1 (2.2)	1 (1.1)
Physical violence (stranger)	37 (27.4)	10 (22.2)	27 (30.3)
Physical violence (acquaintance)	22 (16.3)	7 (15.6)	15 (16.7)
Death of loved one (e.g., homicide)	45 (33.3)	11 (24.4)	34 (37.8)
Serious accident/explosion	40 (29.6)	13 (28.9)	27 (30.7)
Serious illness	88 (65.2)	27 (60.0)	61 (68.5)
Sexual harassment (stranger)	3 (2.2)	0 (0)	3 (3.4)
Sexual harassment (acquaintance)	4 (3.0)	3 (6.7)	1 (1.1)
Neglect	23 (17.0)	5 (11.1)	18 (20.0)
Childhood sexual abuse (stranger)	7 (5.2)	4 (8.9)	3 (3.4)
Childhood sexual abuse (acquaintance)	7 (5.2)	3 (6.7)	4 (4.4)
Natural catastrophe	35 (25.9)	9 (20.0)	26 (28.9)
Other trauma	29 (21.5)	12 (28.6)	17 (20.7)

**Table 5:** Predictors of severity of depressive symptoms in refugee aid workers (PHQ-9 total score; N = 135).

Predictors	B ^d^	95% CI ^e^	SE ^f^	β	*p*-Value
Sociodemographics					
Age	−0.041	−0.087 to 0.006	0.023	−0.160	*p* = 0.084
Gender ^a^	2.438	1.053 to 3.823	0.698	0.315	*p* = 0.001
Migration background ^b^	0.905	−0.565 to 2.376	0.741	0.108	*p* = 0.225
Refugee work related variables					
Type of refugee aid worker (VAD or PAD) ^c^	−1.278	−2.738 to 0.182	0.736	−0.165	*p* = 0.086
Weekly working hours (refugee work)	−0.121	−0.257 to 0.014	0.066	−0.425	*p* = 0.078
Duration of refugee work	−0.0061	−0.029 to 0.018	0.011	−0.081	*p* = 0.620
Burdens of refugee work	0.480	0.188 to 0.773	0.148	0.294	*p* = 0.002
Amount of experienced traumatic events (ETI)	0.698	0.335 to 1.062	0.183	0.357	*p* < 0.001

^a^ 0 = male, 1 = female; ^b^ 0 = no migration background, 1 = migration background; ^c^ 0 = VAD, 1 = PAD; ^d^ B = regression coefficient; ^e^ CI = confidence interval; ^f^ SE = standard error.
